# Dosimetric impacts of endorectal balloon in CyberKnife stereotactic body radiation therapy (SBRT) for early‐stage prostate cancer

**DOI:** 10.1002/acm2.12063

**Published:** 2017-04-13

**Authors:** Hong F. Xiang, Hsiao‐Ming Lu, Jason A. Efstathiou, Anthony L. Zietman, Ricardo De Armas, Kathryn Harris, B. Nicolas Bloch, Muhammad Mustafa Qureshi, Sean Keohan, Ariel E. Hirsch

**Affiliations:** ^1^ Department of Radiation Oncology Boston University School of Medicine and Boston Medical Center Boston Massachusetts USA; ^2^ Department of Radiation Oncology Massachusetts General Hospital and Harvard Medical School Boston Massachusetts USA; ^3^ Massachusetts Institute of Technology Cambridge Massachusetts USA

**Keywords:** CyberKnife, endorectal balloon, prostate cancer, rectum toxicity, SBRT

## Abstract

**Purpose:**

In SBRT for prostate cancer, higher fractional dose to the rectum is a major toxicity concern due to using smaller PTV margin and hypofractionation. We investigate the dosimetric impact on rectum using endorectal balloon (ERB) in prostate SBRT.

**Materials and Methods:**

Twenty prostate cancer patients were included in a retrospective study, ten with ERB and 10 without ERB. Optimized SBRT plans were generated on CyberKnife MultiPlan for 5 × 7.25 Gy to PTV under RTOG‐0938 protocol for early‐stage prostate cancer. For the rectum and the anterior half rectum, mean dose and percentage of volumes receiving 50%, 80%, 90%, and 100% prescription dose were compared.

**Results:**

Using ERB, mean dose to the rectum was 62 cGy (*P *=* *0.001) lower per fraction, and 50 cGy (*P *=* *0.024) lower per fraction for the anterior half rectum. The average V_50%_, V_80%_, V_90%_, and V_100%_ were lower by 9.9% (*P *=* *0.001), 5.3% (*P *=* *0.0002), 3.4% (*P *=* *0.0002), and 1.2% (*P *=* *0.005) for the rectum, and lower by 10.4% (*P *=* *0.009), 8.3% (*P *=* *0.0004), 5.4% (*P *=* *0.0003), and 2.1% (*P *=* *0.003) for the anterior half rectum.

**Conclusions:**

Significant reductions of dose to the rectum using ERB were observed. This may lead to improvement of the rectal toxicity profiles in prostate SBRT.

## 1 Introduction

Based on the evidence of prostate cancer having a relatively low *α*/*β* ratio^1^, ^2^, ^3^ and the significantly improved accuracy in image‐guided target localization and radiation dose delivery,^4^, ^5^, ^6^ hypofractionated stereotactic body radiation therapy (SBRT) for prostate cancer has been investigated at multiple institutions.^7^, ^8^, ^9^, ^10^, ^11^, ^12^ These works have demonstrated that prostate SBRT can result in effective biochemical control while minimizing rectal and bladder toxicities to a level that is comparable to those seen in conventional radiotherapy, including 3D‐CRT(3D Conformal Radiation Therapy), IMRT (Intensity Modulated Radiation Therapy), and HDR (High Dose Rate Brachytherapy). With the follow‐up data approaching 6 years to this date, prostate SBRT has now been considered as an alternative therapeutic option to the conventional radiotherapy for localized prostate cancer, either as a monotherapy for low‐ and intermediate‐risk prostate cancer^13^, ^14^, ^15^, ^16^ or post‐IMRT boost treatment for high‐risk prostate cancer.^17^

In SBRT for prostate cancer, due to the much higher dose per fraction and use of smaller PTV margins (2–3 mm posterior, 3–5 mm in all other directions) than those in 3D‐CRT or IMRT, it is particularly critical to minimize the prostate motion and the exposure of rectum volumes to intermediate and high dose which are predictive factors for late rectal toxicity.^18^, ^19^, ^20^ It has been shown that an air or water‐filled endorectal balloon (ERB) can significantly reduce prostate motion^21^, ^22^, ^23^ and displace the posterior portion of the rectal wall away from the intermediate‐to‐high dose regions in 3D‐CRT and IMRT. This displacement can lead to significant rectal wall sparing and reducing rectal toxicity from prostate or post‐prostatectomy radiation treatment, potentially to allow for further dose escalation to the prostate.^24^, ^25^, ^26^, ^27^, ^28^, ^29^, ^30^

For prostate SBRT, late rectal toxicity data are very limited with maximum follow‐up just under 6 years.^14^, ^15^, ^16^ It can be anticipated that any systematic reduction of rectal dose in such hypofractionated prostate treatment may be beneficial, such as those potentially achievable using ERB to minimize exposing rectal volume to intermediate and high dose. However, to this date, there has been no specific study based on CyberKnife prostate SBRT experience on how using ERB may help to reduce rectum dose and improve rectal dose‐volume profiles under the hypofractionated target dose specifications and OARs (organ‐at‐risk) constraints. In addition, use of ERB has not been included neither on protocols treating prostate alone for early‐stage prostate cancer nor on protocols treating both the prostate and the proximal seminal vesicles for intermediate‐risk and high‐risk prostate cancer.

In this work, we performed a systematic treatment planning study on the potential dosimetric impacts of using ERB for prostate cancer patients who may receive SBRT treatments such as those described in RTOG‐0938 “A Randomized Phase II Trial of Hypo‐fractionated Radiotherapy for Favorable Risk Prostate Cancer”.^31^

## 2 Materials and methods

### 2.A Patient data and use of ERB

**Figure 1:**
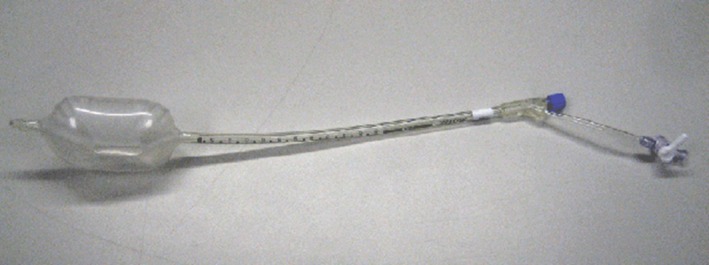
The endorectal balloon (ERB) used for patients included in this planning study.

Twenty prostate cancer patient cases previously treated at two institutions were selected for a retrospective CyberKnife SBRT treatment planning study. Ten of the patients had CT simulation and treatment using ERB filled with 60–100 cc water (the ERB group). The other ten patients had CT simulation and treatment with empty rectum and no ERB (the noERB group). Figure 1 shows the endorectal balloon (ERB) used for patients included in this planning study.

All patients had at least three well‐spaced fiducials implanted in the prostate for image‐guided target volume localization. For patient in the ERB group, immediately before CT simulation scan, an indexed lumen ERB (RadiaDyne, LLC, Houston, TX, USA) was inserted into the rectum, filled with 60–100 cc water so that their density would be essentially homogeneous with that of the surrounding tissue. For patients in the noERB group, instructions were given for pre‐CT and pretreatment bowel preparation, including dietary guidelines, use of anti‐gas tablets, and administered enemas to ensure an empty rectum.

### 2.B SBRT planning and dosimetric comparison

The gross tumor volume (GTV) for this study refers to the prostate alone per RTOG‐0938 protocol for early‐stage prostate cancer. The clinical target volume (CTV) was the same as the GTV consisting of the prostate alone without including the seminal vesicles. Prostates were drawn by two radiation oncologists using the noncontrast axial CT scans for planning. The planning target volume (PTV) was defined as the CTV plus a 3 mm margin posteriorly and 5 mm in all other directions. Normal tissue organs such as bladder and rectum were contoured as solid organs instead of contouring the bladder and rectal walls. The bladder was contoured from its base to the dome. The rectum was contoured from the anus at the level of the ischial tuberosities to the recto‐sigmoid flexure, generally below the bottom of the sacroiliac joints. Because the anterior half of the rectum is in touch or in overlap with the prostate PTV and more subject to intermediate‐to‐high dose, it was contoured by bisecting the rectum contour into the anterior half and the posterior half slice‐by‐slice and evaluated separately for comparison.

An optimized SBRT plan was generated for each case using the multi‐objective sequential optimization in CyberKnife MultiPlan TPS system to meet the five fraction (5 × 725 cGy) dose‐specification and dose‐volume constraints per RTOG‐0938 for early‐stage prostate cancer. These plans typically used 2–3 collimators of different sizes, and 100–200 noncoplanar and nonisocentric beams of 6 MV x ray. Target dose coverage was characterized by the new conformity index (nCI), heterogeneity index, and mean PTV dose. Plans were typically prescribed to 79%–85% isodose line (IDL) to ensure that at least 95% of the PTV was covered by the prescription dose. Dosimetric parameters for the rectum and the anterior half of the rectum were compared between the two groups, including the mean dose and the percentages of volume receiving 50%, 80%, 90%, and 100% of the prescription dose.

### 2.C Statistical analysis

Descriptive statistics were calculated for each dosimetric parameter in the ERB group and noERB group. Mean and standard deviation are reported along with the difference in mean value for each parameter and denoted as Δ. Independent samples *t*‐tests were performed to examine the differences between the two groups. The analyses were repeated after logarithmic (lg 10) transformation of the dosimetric data. All statistical analyses were performed using the SPSS software for Windows (version 20.0, SPSS Inc., Chicago, IL, USA). Differences with *P*‐value <0.05 was considered statistically significant.

## 3 Results

**Figure 2:**
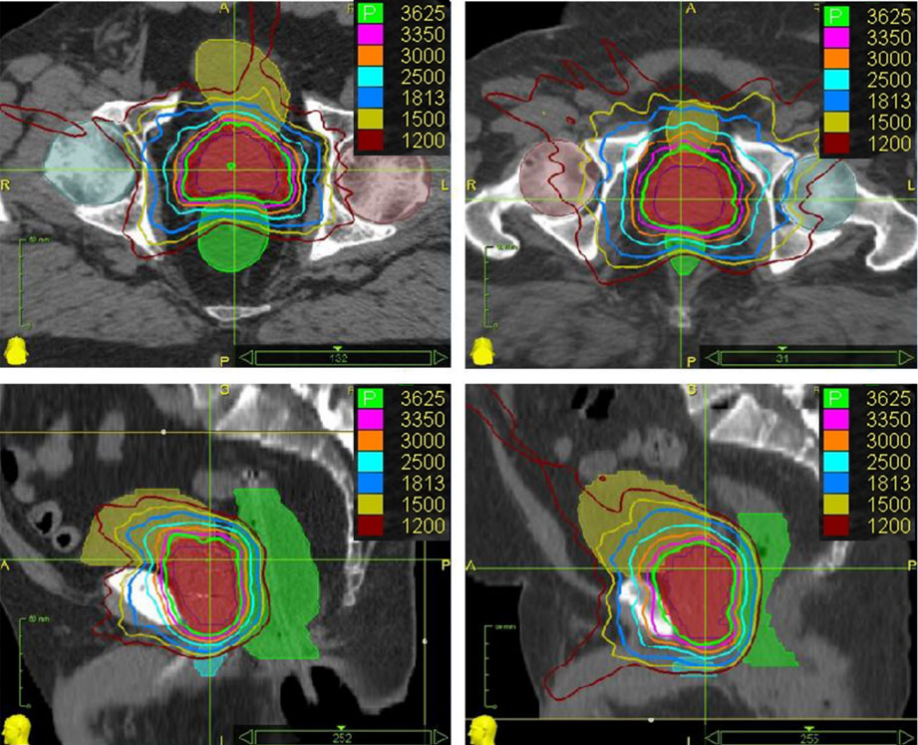
Typical dose distributions of CyberKnife prostate SBRT plans for a case from the ERB group (left) and a case from the noERB group (right). The top row for axial view, bottom row for sagittal view. The anterior half of the rectum contours were obtained by bisecting the full rectum contours from the midline slice‐by‐slice.

Figure 2 shows the isodose distributions for a case from the ERB group (left) and a case from the noERB group (right). It demonstrates that rectal volumes receiving intermediate‐to‐high dose (18.3–36.25 Gy, i.e., 50–100% of the prescription dose 36.25 Gy) was reduced using ERB.

**Figure 3:**
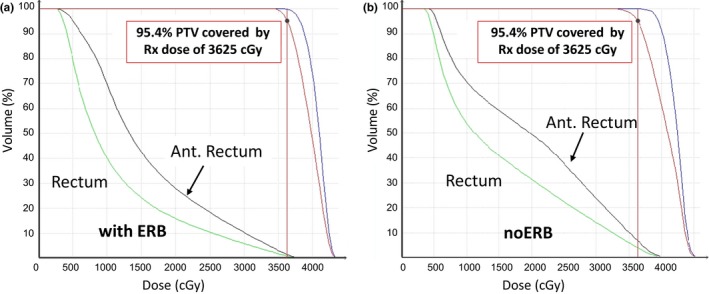
DVHs for a typical case in the ERB group (a) and in the noERB group (b).

Figure 3 shows the dose‐volume histograms (DVHs) for rectum and the anterior half rectum in the two typical cases, with the ERB case (left: a) having lower profiles than the noERB case (right: b). Blue for prostate, red for PTV, green for rectum, and black for the anterior half of the rectum. This rectal sparing effect is similar to what was reported in 3D‐CRT and IMRT studies using ERB, which was caused by the expansion of the whole rectum and the displacement of the rectum walls laterally and posteriorly away from the intermediate‐to‐high dose region.

Table 1 shows the plan dosimetric characteristics for the ERB group versus noERB group, including the volumes of the prostate (CTV) and PTV, mean dose to PTV, PTV volume coverage in % by prescription dose, dose conformity index, dose heterogeneity index, and plan prescription isodose line. For target, all cases have at least 95% of the PTV covered by prescription dose of 36.25 Gy, with median of the PTV mean dose at 39.9 Gy (range 38.9–40.8 Gy) for the ERB group, and median of the PTV mean dose at 39.8 Gy (range 39.2–40.6 Gy) for the noERB group. Overall, target volumes and their dosimetric characteristics are comparable between the two groups.

**Table 1:** PTV dosimetric characteristics for the ERB group versus noERB group

Volumes	ERB Group (*N *= 10)		noERB Group (*N *= 10)	
	Median	Range	Median	Range
Prostate (cc)	40.0	16.6–87.4	44.7	22.8–87.1
PTV (cc)	79.3	41.6–151.5	80.7	48.3–143.7
Conformality Index	1.18	1.11–1.25	1.18	1.09–1.28
Heterogeneity Index	1.23	1.18–1.25	1.23	1.20–1.27
Rx Isodose Line	81%	80%–85%	81%	79%–83%
PTV coverage	96%	95%–98%	96%	95%–97%
PTV mean dose (cGy)	3987	3893–4077	3982	3915–4062

ERB, Endorectal balloon; N, number of patients; PTV, planning target volume; Rx, prescription.

Table 2 shows the comparison of the dosimetric parameters for rectum volumes and the anterior half rectum volumes between the ERB group and the noERB group. V_x%_, (x% = 50%, 80%, 90%, 100%) represent the percentage volumes exposed to x% of the prescription dose (36.25 Gy) for the rectum volumes and the anterior half rectum volumes.

**Table 2:** Comparison of the dosimetric characteristics for the rectum and the anterior half rectum volumes

Volumes	DVH Metrics	ERB Group (*N *=* *10)		noERB Group (*N *=* *10)		∆	*P*‐Value
		Mean ± SD	(Range)	Mean ± SD	(Range)		
Rectum	Mean dose (Gy)	10.4 ± 1.8	8.2–13.5	13.5 ± 1.9	11.1–16.6	3.1	0.001
	V_50%_ (%)	18.1 ± 4.8	12.0–24.8	28.0 ± 6.8	18.1–40.7	9.9	0.001
	V_80%_ (%)	6.9 ± 1.9	3.8–9.2	12.2 ± 3.2	7.5–16.8	5.3	0.0002
	V_90%_ (%)	3.9 ± 1.1	1.9–5.2	7.3 ± 2.0	3.7–9.5	3.4	0.0002
	V_100%_ (%)	1.0 ± 0.4	0.4–1.7	2.2 ± 1.1	0.7–3.9	1.1	0.005
Anterior rectum wall	Mean dose (Gy)	14.9 ± 2.5	11.6–19.6	17.4 ± 2.0	14.7–20.2	2.5	0.024
	V_50%_ (%)	33.0 ± 8.7	23.3–48.6	43.4 ± 7.2	33.2–54.2	10.4	0.009
	V_80%_ (%)	12.7 ± 3.4	7.8–17.9	21.0 ± 5.0	14.0–28.0	8.3	0.0004
	V_90%_ (%)	7.2 ± 1.9	3.8–9.9	12.6 ± 3.4	6.4–16.9	5.4	0.0003
	V_100%_ (%)	1.8 ± 0.6	1.0–2.8	3.9 ± 1.9	1.2–6.8	2.1	0.003

DVH, Dose‐volume histogram; ERB, Endorectal balloon; N, number of patients; SD, standard deviation; Δ, Difference between mean noERB and ERB values.

As shown in Table 2, mean dose to the rectum was significantly lower for the ERB group (mean 10.4 Gy) than the noERB group (mean 13.5 Gy), an average reduction of 3.1 Gy (*P *=* *0.001), or 62 cGy lower per fraction (*P *=* *0.001). Similarly, mean dose to the anterior half rectum was also significantly lower for the ERB group (mean 14.9 Gy) than the noERB Group (mean 17.4 Gy), an average reduction of 2.5 Gy ((*P *=* *0.024), or 50 cGy lower per fraction (*P *=* *0.024).

A pattern of significant reduction in rectum volumes (in percentage) receiving 50%, 80%, 90%, and 100% of prescription dose (36.25 Gy) was seen between the ERB Group and the noERB Group. The average V_50%,_ V_80%,_ V_90%_, and V_100%_ for the ERB Group were 18.1%, 6.9%, 3.9%, and 1.0% in comparison to 28.0%, 12.2%, 7.3%, and 2.2% for the noERB group, a reduction of 9.9% (*P *=* *0.001), 5.3% (*P *=* *0.0002), 3.4% (*P *=* *0.0002), and 1.1% (*P *=* *0.005), respectively.

Further, a pattern of more notable reduction in anterior half rectum volumes (in percentage) receiving 50%, 80%, 90%, and 100% of prescription dose (36.25 Gy) was observed between the ERB Group and the noERB Group. The average V_50%,_ V_80%,_ V_90%_, and V_100%_ for the ERB Group were 33.0%, 12.7%, 7.2%, and 1.8% in comparison to 43.4%, 21.0%, 12.6%, and 3.9% for the noERB group, a significant reduction of 10.4% (*P *=* *0.009), 8.3% (*P *=* *0.0004), 5.4% (*P *=* *0.0003), and 2.1% (*P *=* *0.003), respectively.

Similar results were obtained when the analyses were repeated on logarithmic‐transformed data. All the above comparisons remained statistically significant (data not shown).

**Figure 4:**
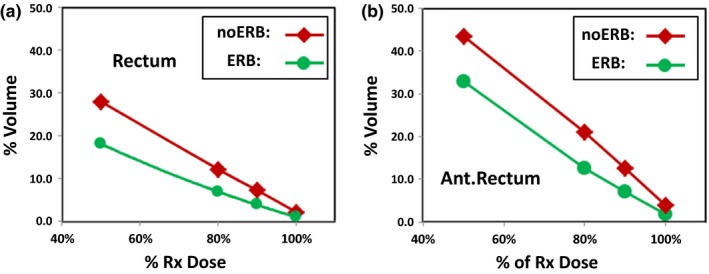
DVHs profiles for the rectum volume (left) and anterior half rectum volume (right) in the intermediate‐to‐high dose region 50%–100% of prescription dose 36.25 Gy.

Figure 4 shows the DVH profile comparison between the ERB group and the noERB group for the rectum (left) and the anterior half rectum (right) in the intermediate‐to‐high dose region for the prescription dose of 36.25 Gy (5 × 7.25 Gy). Data points are the averaged percentage volumes at 50%, 80%, 90%, and 100% of the prescription dose.

## 4 Discussion

### 4.A Significance of rectum dose reduction for SBRT

Using ERB to reduce dose to anorectal wall has been reported on 3D‐CRT and IMRT for prostate cancer. Patel et al. 30 first reported that using ERB in 3D‐CRT of 38 × 2.0 Gy led to significant high‐dose rectal sparing comparable to that achieved by a highly conformal IMRT of 38 × 2.0 Gy. Further sparing can be achieved in IMRT using rectal balloon. An overall average rectal sparing ratio (RSR) of 0.61, that is, a mean fractional high‐dose rectal sparing of 39%, was reported for rectal volumes receiving ≥65 Gy (RSR is defined as rectum's high dose volume with ERB inflated, divided by the volume with ERB deflated). VanLin et al. 29 reported that in both 3D‐CRT and IMRT, using ERB can lead to significant dose reduction for rectum exposed to intermediate and high dose. Smeenk et al. 27 reported a 12 Gy mean dose reduction for the anal wall in 3D‐CRT of 39 × 2 Gy, and a 7.5 Gy mean dose reduction in IMRT of 39 × 2 Gy.

This study is the first, to the best of our knowledge, to show that a systematic dose reduction effect also exists for CyberKnife‐based prostate SBRT under the extreme hypofractionation of 5 × 7.25 Gy for treating early‐stage prostate cancer. Due to the much higher dose per fraction, the magnitude of per fraction rectal dose reduction in SBRT may bear more significance in relation to both the acute and the late rectal toxicity than those seen for 3D‐CRT or IMRT. In terms of absolute dose, using ERB in SBRT of 5 × 7.25 Gy was shown to have an average 3.1 Gy lower mean dose to the rectum, and an average 2.5 Gy lower mean dose to the anterior half rectum. This appears to be smaller magnitude of reduction than the 7.5–12.0 Gy mean dose reduction seen in 3D‐CRT and IMRT of 39 × 2.0 Gy.^27^ However, in terms of fractional dose reduction, the 2.5–3.1 Gy overall reduction in SBRT is 50–60 cGy reduction per fraction; this is a much higher dose reduction per fraction than those seen in 3D‐CRT and IMRT of 20–30 cGy dose reduction per fraction.^27^

The rectum dose reduction we have seen between patients planned with ERB and noERB is a meaningful finding in the context of extreme hypofractionated CyberKnife prostate SBRT of 36.25 Gy in five fractions. These results and the effective plan optimization technique may turn out to be even more important in view of the more recent interests in prostate focal therapy approach by generating a simultaneously integrated dose escalation to 47.5 Gy onto the dominant intra‐prostatic lesion while maintaining the 36.25 Gy to the prostate gland.^32^, ^33^

A recent study by Wong, et al. reported an increase in the volume of rectum and rectal wall receiving high‐dose radiation using an ERB during SBRT in the form of RapidArc volumetric modulated arc therapy (VMAT) using 6 MV flattering filter‐free photon beams. This observation differs from the results in our study using 6 MV photon beams on CyberKnife for SBRT, as well as the general rectum dose reductions seen in previous studies with 3D‐CRT and IMRT.^27^, ^29^, ^30^ This difference may be related to two important points emphasized in our study. First, in planning, we strictly followed the RTOG 0938 planning DVHs constraint guidelines, especially the DVH constraints for rectal volumes receiving high doses, V3806 < 1 cc (3806 cGy corresponding to 105% of the prescription dose of 3625 cGy) and V3440 < 3 cc (corresponding to 95% of the prescription dose). Our results showed similar V100% of both the rectum volume and the anterior rectum volume for the ERB group vs. the noERB group, as revealed in Fig. 4. Second, the use of non‐coplanar and non‐isocentric beams from CyberKnife physically offered a better rectal sparing dosimetry advantage as there are no posterior beams going through rectum, and there are only anterior and anterior oblique beamlets in CyberKnife‐based SBRT in comparison to the beams used in gantry‐based RapidArc(VMAT) SBRT.

### 4.B On the study design regarding patient data group selection

It should be pointed out that unlike those done in previous studies for 3D‐CRT and IMRT, as well as the recent study for SBRT by Wong, et al.,^34^ patient data included in this study were not repeated CT scans of same group of patients with ERB versus noERB. Theoretically, a more rigorous controlled comparison would be helpful to provide direct insight by selecting the same group of patients, with repeated CT scans once with ERB and once without ERB. Unfortunately, at the start of this study, the potential dosimetric benefit of using ERB was not clear because of the lacking of such comparison data for prostate SBRT.

Instead, we collected data from two separate patient groups: one used EBR, and the other did not use ERB per institution protocol acceptance. We chose to compare the two groups’ dosimetric profiles for the percentage rectum volumes receiving intermediate‐to‐high doses. This approach echo the general methodology used in randomized clinical trials where two patient groups of the same disease profile were randomized to go through different treatment schemes for comparison.

Our study intent is to examine the general profiles of the rectum mean dose and dose‐volume histograms of the two patient groups, though not ideally having each patient's consecutive CT scans with one having ERB and the other with no ERB. Nevertheless, as shown in Table 1, the two groups have similar target volume and dosimetry characteristics, and were both planned under the dose‐specification and dose‐volume constraints of a common SBRT protocol RTOG 0938^31^ of hypofractionated RT for early‐stage prostate cancer. Therefore, our approach also provides clinically relevant and useful comparison.

### 4.C Rectum DVHs and toxicity profiles in prostate SBRT

Recently, King et al. reviewed patient health related QoL (Quality‐of‐Life) follow‐up after SBRT for 864 patients of localized prostate cancer.^15^ Rectum toxicity profiles at median follow‐up of 3 yr for late grade 3 GI (gastrointestinal) toxicities typically lies within the 1%–3% range, which is comparable to those seen in conventionally fractionated 3D‐CRT, IMRT, or HDR. The rectum DVH objectives in these prostate SBRT are generally V_50%_ <50% (rectum volume receiving 50% of the prescribed dose is <50%), V_80%_ <20%, V_90%_ <10%, and V_100%_ <5%.^11^, ^12^, ^15^, ^16^ Our study demonstrated a pattern of systematic improvements in DVH profiles of the rectum using ERB as seen in Table 2, Figs. 3 and 4. The rectum DVHs for both the ERB and the noERB group are much better than the general objectives in the review.^15^ In addition, we also examined the DVH profiles for the anterior half rectum volumes. The exposure of the anterior half rectum to intermediate‐to‐high dose bears more relevance in evaluating rectal toxicity in SBRT.

Unlike in IMRT or 3D‐CRT of 79.2 Gy where a 15% rectum volume receiving dose greater than 70 Gy is known to be an independent predictor for late grade 2 rectal toxicity,^20^ there has been no established upper limit in prostate SBRT for rectum DVHs based on analyzing clinical data in the intermediate‐to‐high dose region. In evaluating prostate SBRT treatment and rectal toxicity, mean dose and DVHs profiles of rectum, especially the anterior half of the rectum, should be minimized to ALARA (As Low As Reasonably Achievable) while respecting the relatively general objectives for rectum DVHs.^15^ Overall, this study showed systematically better DVH profiles for rectum using ERB. Potentially, such dosimetric improvements may lead to better rectal toxicity profiles for SBRT treatment; yet, this hypothesis remains to be examined with clinical follow‐up data when it becomes available.

It should be noted that this study is limited to SBRT planning for early‐stage prostate cancer where the CTV is defined as prostate volume alone without including the seminal vesicles (SV). This may explain why in general we achieved overall much better rectum DVH profiles (as seen in Table 2 and Fig. 3) than those described by King et al.^15^ This can be due to the fact that smaller section of the rectum is involved when CTV includes prostate alone. Patel et al.^30^ reported significant rectal sparing using ERB in IMRT for five patients with and without inclusion of seminal vesicles. In comparison, they observed significant (about 10% more) rectal sparing in terms of reducing rectum volumes receiving intermediate‐to‐high dose of 55–70 Gy for plans with prostate alone in CTV than those with both prostate and SV in CTV. As a next step, we will extend our analysis to cases of intermediate risk and high risk by including the proximal section of the seminal vesicles into the CTV to characterize the potential dosimetry improvement using ERB.

## 5 Conclusions

In conclusion, significant reductions of dose to the rectum using ERB were observed in the intermediate and high‐dose region from a retrospective planning study of CyberKnife prostate SBRT. This may be considered as a valuable technique for clinical implementation to improve the rectal toxicity profiles in prostate SBRT.

## Conflict of Interest

The authors have no conflict of interest to report.

## References

[1] Brenner DJ , Hall EJ . Fractionation and protraction for radiotherapy of prostate carcinoma. Int J Radiat Oncol Biol Phys. 1999;43:1095–1101. 10192361 10.1016/s0360-3016(98)00438-6

[2] Fowler JF . The radiobiology of prostate cancer including new aspects of fractionated radiotherapy. Acta Oncol. 2005;44:265–276. 16076699 10.1080/02841860410002824

[3] Miralbell R , Roberts SA , Zubizarreta E , Hendry JH . Dose‐fractionation sensitivity of prostate cancer deduced from radiotherapy outcomes of 5,969 patients in seven international institutional datasets: Alpha/beta = 1.4 (0.9–2.2) Gy. Int J Radiat Oncol Biol Phys. 2012;82:e17–e24. 21324610 10.1016/j.ijrobp.2010.10.075

[4] Xie Y , Djajaputra D , King CR , Hossain S , Ma L , Xing L . Intrafractional motion of the prostate during hypofractionated radiotherapy. Int J Radiat Oncol Biol Phys. 2008; 72:236–246. 18722274 10.1016/j.ijrobp.2008.04.051 PMC2725181

[5] Langen KM , Willoughby TR , Meeks SL , et al. Observations on real‐time prostate gland motion using electromagnetic tracking. Int J Radiat Oncol Biol Phys. 2008;71:1084–1090. 18280057 10.1016/j.ijrobp.2007.11.054

[6] De Los Santos J , Popple R , Agazaryan N , et al. Image guided radiation therapy (IGRT) technologies for radiation therapy localization and delivery. Int J Radiat Oncol Biol Phys. 2013;87:33–45. 23664076 10.1016/j.ijrobp.2013.02.021

[7] Madsen BL , Hsi RA , Pham HT , Fowler JF , Esagui L , Corman J . Stereotactic hypofractionated accurate radiotherapy of the prostate (SHARP), 33.5 Gy in five fractions for localized disease: First clinical trial results. Int J Radiat Oncol Biol Phys. 2007;67:1099–1105. 17336216 10.1016/j.ijrobp.2006.10.050

[8] King CR , Brooks JD , Gill H , Pawlicki T , Cotrutz C , Presti JC Jr . Stereotactic body radiotherapy for localized prostate cancer: Interim results of a prospective phase II clinical trial. Int J Radiat Oncol Biol Phys. 2009;73:1043–1048. 18755555 10.1016/j.ijrobp.2008.05.059

[9] Katz AJ , Santoro M , Ashley R , Diblasio F , Witten M . Stereotactic body radiotherapy for organ‐confined prostate cancer. BMC urology. 2010;10:1. 20122161 10.1186/1471-2490-10-1 PMC2831888

[10] Freeman DE , King CR . Stereotactic body radiotherapy for low‐risk prostate cancer: Five‐year outcomes. Radiation oncology. 2011;6:3. 21219625 10.1186/1748-717X-6-3 PMC3022740

[11] McBride SM , Wong DS , Dombrowski JJ , et al. Hypofractionated stereotactic body radiotherapy in low‐risk prostate adenocarcinoma: Preliminary results of a multi‐institutional phase 1 feasibility trial. Cancer. 2012;118:3681–3690. 22170628 10.1002/cncr.26699

[12] King CR , Brooks JD , Gill H , Presti JC Jr . Long‐term outcomes from a prospective trial of stereotactic body radiotherapy for low‐risk prostate cancer. Int J Radiat Oncol Biol Phys. 2012;82:877–882. 21300474 10.1016/j.ijrobp.2010.11.054

[13] ASTRO Model Policies . Stereotactic Body Radiation Therapy (SBRT). https://www.astro.org/uploadedFiles/Main_Site/Practice_Management/Reimbursement/2013HPcoding%20guidelines_SBRT_Final.pdf. Accessed 01 March 2017.

[14] King CR , Freeman D , Kaplan I , et al. Stereotactic body radiotherapy for localized prostate cancer: Pooled analysis from a multi‐institutional consortium of prospective phase II trials. Radiother Oncol. 2013;109:217–221. 24060175 10.1016/j.radonc.2013.08.030

[15] King CR , Collins S , Fuller D , et al. Health‐related quality of life after stereotactic body radiation therapy for localized prostate cancer: Results from a multi‐institutional consortium of prospective trials. Int J Radiat Oncol Biol Phys. 2013;87:939–945. 24119836 10.1016/j.ijrobp.2013.08.019

[16] Katz AJ , Santoro M , Diblasio F , Ashley R . Stereotactic body radiotherapy for localized prostate cancer: Disease control and quality of life at 6 years. Radiation oncology. 2013;8:118. 23668632 10.1186/1748-717X-8-118 PMC3674983

[17] Jabbari S , Weinberg VK , Kaprealian T , et al. Stereotactic body radiotherapy as monotherapy or post‐external beam radiotherapy boost for prostate cancer: Technique, early toxicity, and PSA response. Int J Radiat Oncol Biol Phys. 2012;82:228–234. 21183287 10.1016/j.ijrobp.2010.10.026

[18] D'Amico AV , Manola J , McMahon E , et al. A prospective evaluation of rectal bleeding after dose‐escalated three‐dimensional conformal radiation therapy using an intrarectal balloon for prostate gland localization and immobilization. Urology. 2006;67:780–784. 16584760 10.1016/j.urology.2005.10.008

[19] Tucker SL , Zhang M , Dong L , Mohan R , Kuban D , Thames HD . Cluster model analysis of late rectal bleeding after IMRT of prostate cancer: A case‐control study. Int J Radiat Oncol Biol Phys. 2006;64:1255–1264. 16504763 10.1016/j.ijrobp.2005.10.029

[20] Michalski JM , Yan Y , Watkins‐Bruner D , et al. Preliminary toxicity analysis of 3‐dimensional conformal radiation therapy versus intensity modulated radiation therapy on the high‐dose arm of the radiation therapy oncology group 0126 prostate cancer trial. Int J Radiat Oncol Biol Phys. 2013;87:932–938. 24113055 10.1016/j.ijrobp.2013.07.041 PMC3840044

[21] Both S , Wang KK , Plastaras JP , et al. Real‐time study of prostate intrafraction motion during external beam radiotherapy with daily endorectal balloon. Int J Radiat Oncol Biol Phys. 2011;81:1302–1309. 21035952 10.1016/j.ijrobp.2010.08.052

[22] Smeenk RJ , Louwe RJ , Langen KM , et al. An endorectal balloon reduces intrafraction prostate motion during radiotherapy. Int J Radiat Oncol Biol Phys. 2012;83:661–669. 22099035 10.1016/j.ijrobp.2011.07.028

[23] Wang KK , Vapiwala N , Deville C , et al. A study to quantify the effectiveness of daily endorectal balloon for prostate intrafraction motion management. Int J Radiat Oncol Biol Phys. 2012;83:1055–1063. 22115790 10.1016/j.ijrobp.2011.07.038

[24] Deville C , Both S , Bui V , et al. Acute gastrointestinal and genitourinary toxicity of image‐guided intensity modulated radiation therapy for prostate cancer using a daily water‐filled endorectal balloon. Radiation oncology. 2012;7:76. 22621764 10.1186/1748-717X-7-76 PMC3464898

[25] Smeenk RJ , Van Lin EN , Van Kollenburg P , McColl GM , Kunze‐Busch M , Kaanders JH . Endorectal balloon reduces anorectal doses in post‐prostatectomy intensity‐modulated radiotherapy. Radiother Oncol. 2011;101:465–470. 21872953 10.1016/j.radonc.2011.07.019

[26] Smeenk RJ , Teh BS , Butler EB , Van Lin EN , Kaanders JH . Is there a role for endorectal balloons in prostate radiotherapy? A systematic review. Radiother Oncol. 2010;95:277–282. 20451274 10.1016/j.radonc.2010.04.016

[27] Smeenk RJ , Van Lin EN , Van Kollenburg P , Kunze‐Busch M , Kaanders JH . Anal wall sparing effect of an endorectal balloon in 3D conformal and intensity‐modulated prostate radiotherapy. Radiother Oncol. 2009;93:131–136. 19523704 10.1016/j.radonc.2009.05.014

[28] Vargas C , Mahajan C , Fryer A , et al. Rectal dose‐volume differences using proton radiotherapy and a rectal balloon or water alone for the treatment of prostate cancer. Int J Radiat Oncol Biol Phys. 2007;69:1110–1116. 17967305 10.1016/j.ijrobp.2007.04.075

[29] Van Lin EN , Hoffmann AL , Van Kollenburg P , Leer JW , Visser AG . Rectal wall sparing effect of three different endorectal balloons in 3D conformal and IMRT prostate radiotherapy. Int J Radiat Oncol Biol Phys. 2005;63:565–576. 16168848 10.1016/j.ijrobp.2005.05.010

[30] Patel RR , Orton N , Tome WA , Chappell R , Ritter MA . Rectal dose sparing with a balloon catheter and ultrasound localization in conformal radiation therapy for prostate cancer. Radiother Oncol. 2003;67:285–294. 12865176 10.1016/s0167-8140(03)00056-2

[31] NRG Oncology RTOG 0938 . A Randomized Phase II Trial of Hypofractionated Radiotherapy for Favorable Risk Prostate Cancer. https://www.rtog.org/ClinicalTrials/ProtocolTable/StudyDetails.aspx?study=0938. Accessed 01 March 2017.

[32] Xiang HF , Cheng J , Alshammari M , et al. Dosimetric validation of robotic prostate SBRT with simultaneous integrated dose escalation to dominant intraprostatic lesion using a magnetic resonance‐based 3D‐printed prostate model in an anthropomorphic pelvis phantom. Int J Radiat Oncol Biol Phys. 2016;96:E613 (manuscript in submission, January, 2017)

[33] Tree A , Jones C , Sohaib A , Khoo V , Van As N . Prostate stereotactic body radiotherapy with simultaneous integrated boost: Which is the best planning method? Radiation Oncology. 2013;8:228. 24088319 10.1186/1748-717X-8-228 PMC3853231

[34] Wong AT , Schreiber D , Agarwal M , Polubarov A , Schwartz D . Impact of the use of an Endorectal balloon on rectal dosimetry during stereotactic body radiation therapy for localized prostate cancer. Practical Radiation Oncology. 2016;6:262–267. 26725959 10.1016/j.prro.2015.10.019

